# Vedolizumab for prevention of lower-GI acute GVHD in the Japanese subgroup analysis of the phase 3 GRAPHITE study

**DOI:** 10.1007/s12185-025-03955-9

**Published:** 2025-03-12

**Authors:** Tatsunori Goto, Hiroshi Okamura, Takashi Ikeda, Yasuo Mori, Souichi Shiratori, Shin-ichiro Fujiwara, Noriko Doki, Ken-ichi Matsuoka, Yuta Katayama, Yi-Bin Chen, Yngvar Fløisand, Guillermo Rossiter, Johan Jansson, Ryou Nakaya, Takanori Teshima

**Affiliations:** 1 Department of Hematology, Japanese Red Cross Aichi Medical Center Nagoya Daiichi Hospital, Nagoya, Japan; 2 Department of Hematology, Graduate School of Medicine, Osaka Metropolitan University, Osaka, Japan; 3 Division of Hematology and Stem Cell Transplantation, Shizuoka Cancer Center, Shizuoka, Japan; 4 Department of Hematology, Oncology and Cardiovascular Medicine, Kyushu University Hospital, Fukuoka, Japan; 5 Department of Hematology, Hokkaido University Faculty of Medicine, Sapporo, Japan; 6 Division of Hematology, Jichi Medical University, Tochigi, Japan; 7 Hematology Division, Tokyo Metropolitan Cancer and Infectious Diseases Center, Komagome Hospital, Tokyo, Japan; 8 Department of Hematology and Oncology, Okayama University Hospital, Okayama, Japan; 9 Department of Hematology, Hiroshima Red Cross Hospital & Atomic-Bomb Survivors Hospital, Hiroshima, Japan; 10 Hematopoietic Cell Transplant and Cellular Therapy Program, Massachusetts General Hospital, Boston, MA USA; 11 Centre for Cancer Cell Reprogramming, Institute of Clinical Medicine, Faculty of Medicine, University of Oslo, 0379 Oslo, Norway; 12 Takeda Development Center Americas, Inc. (at the time of the study), Cambridge, MA USA; 13 Takeda Pharmaceutical Company Limited, Tokyo, Japan; 14 Present Address: Department of Hematology, Endocrinology and Metabolism, Institute of Biomedical Sciences, Tokushima University Graduate School, Tokushima, Japan

**Keywords:** Randomized controlled trial, Vedolizumab, Lower GI, Graft-versus-host disease, Allo-HSCT

## Abstract

In the randomized, double-blind, phase 3 GRAPHITE study (NCT03657160), anti-α_4_β_7_ integrin antibody vedolizumab showed greater efficacy than placebo for prevention of lower-gastrointestinal (GI) acute graft-versus-host disease (aGVHD) after unrelated allogenic hematopoietic stem cell transplantation (allo-HSCT). This post hoc analysis assessed the efficacy and safety of vedolizumab versus placebo for lower-GI aGVHD prevention in Japanese and non-Japanese patients, when added to standard GVHD prophylaxis (calcineurin inhibitor + methotrexate/mycophenolate mofetil + / − anti-thymocyte globulin [ATG]). The analysis included 35 (18 vedolizumab-treated, 17 placebo-treated) Japanese and 298 (150 vedolizumab-treated, 148 placebo-treated) non-Japanese patients. Lower-GI aGVHD-free survival by day + 180 after allo-HSCT (primary endpoint) was 94% in vedolizumab-treated versus 81% in placebo-treated Japanese patients (HR 0.36; 95% CI 0.03–4.01; P = 0.2) and 84% in vedolizumab-treated versus 70% in placebo-treated non-Japanese patients (HR 0.47; 95% CI 0.28–0.78; P = 0.002). The number of events for the 5 key secondary endpoints (lower-GI aGVHD-free and relapse-free survival, Grade C–D aGVHD-free survival, non-relapse mortality, overall survival, and Grade B–D aGVHD-free survival) by day + 180 was lower in vedolizumab- versus placebo-treated Japanese patients. No safety concerns were identified for vedolizumab use as lower-GI aGVHD prophylaxis in Japanese patients.

**Supplementary Information:**

The online version contains supplementary material available at 10.1007/s12185-025-03955-9.

## Introduction

Acute graft-versus-host disease (aGVHD) remains a major cause of morbidity and mortality after allogeneic hematopoietic stem cell transplantation (allo-HSCT), with approximately 40–50% of allo-HSCT recipients developing Grade 2–4 aGVHD, despite routine prophylaxis [1, 2]. Evidence suggests that lower-gastrointestinal (GI) aGVHD is the main driver of aGVHD-associated mortality [3, 4]. The pathophysiology of lower-GI aGVHD involves the stimulation of allogeneic donor T cells by recipient allo-antigens, resulting in the migration of T cells, macrophages, and neutrophils to the intestinal mucosa [5]. Effective prophylactic strategies for lower-GI aGVHD are limited, although preclinical modeling of allogeneic bone marrow transplantation [6, 7], together with imaging studies [8], have suggested that the gut invasion of allo-reactive donor T cells might be blocked by disrupting the interaction of α_4_β_7_ integrin with its adhesion molecule ligand, mucosal vascular addressin cell adhesion molecule 1, localized on the vascular endothelium.

Vedolizumab is a gut-selective anti-lymphocyte trafficking antibody that binds to α_4_β_7_ integrin and blocks its signaling with mucosal vascular addressin cell adhesion molecule 1. This prevents α_4_β_7_-expressing T cells from infiltrating gut tissue, thereby reducing GI tract inflammation [9, 10]. The efficacy of vedolizumab for the treatment of inflammatory bowel diseases such as Crohn’s disease, ulcerative colitis, and pouchitis has been demonstrated [11–21], and vedolizumab is approved for the treatment of moderate to severe Crohn’s disease and ulcerative colitis in over 70 countries worldwide, including Japan [22, 23]. Evidence from preclinical studies [6–8] first indicated that vedolizumab inhibition of the homing of alloreactive T cells and transmigration to GI lymphoid tissue might also be effective for the prevention of lower-GI aGVHD after allo-HSCT [24]. Vedolizumab (300 mg, intravenous [IV]) was well tolerated when added to conventional GVHD prophylaxis in a phase 1b study of adults undergoing allo-HSCT, with a low incidence of overall and lower-GI aGVHD observed [25]. In addition, there have been promising reports on the use of vedolizumab for the treatment of lower-GI aGVHD after allo-HSCT in pediatric patients in Japan [26, 27] and in adults and pediatric patients in other countries [28–30].

The GRAPHITE study was a randomized, double-blind, placebo-controlled, multicenter, phase 3 trial (NCT03657160; EudraCT number, 2018–002141-11), which evaluated the efficacy and safety of IV vedolizumab for the prevention of lower-GI aGVHD, when added to standard GVHD prophylaxis with a calcineurin inhibitor and either methotrexate (MTX) or mycophenolate mofetil (MMF) with or without anti-thymocyte globulin (ATG) in patients undergoing unrelated allo-HSCT as treatment for a hematologic malignancy or myeloproliferative disorder [31]. The results from this international study demonstrated that vedolizumab was more effective than placebo for the prevention of lower-GI aGVHD after allo-HSCT.

Here, we present the efficacy and safety of IV vedolizumab for the prevention of lower-GI GVHD in a subgroup of Japanese and non-Japanese unrelated allo-HSCT recipients, assessed as a post hoc analysis of the GRAPHITE clinical trial, with follow-up for 1 year after transplant.

## Materials and methods

### Study design and medications

**Fig. 1:**
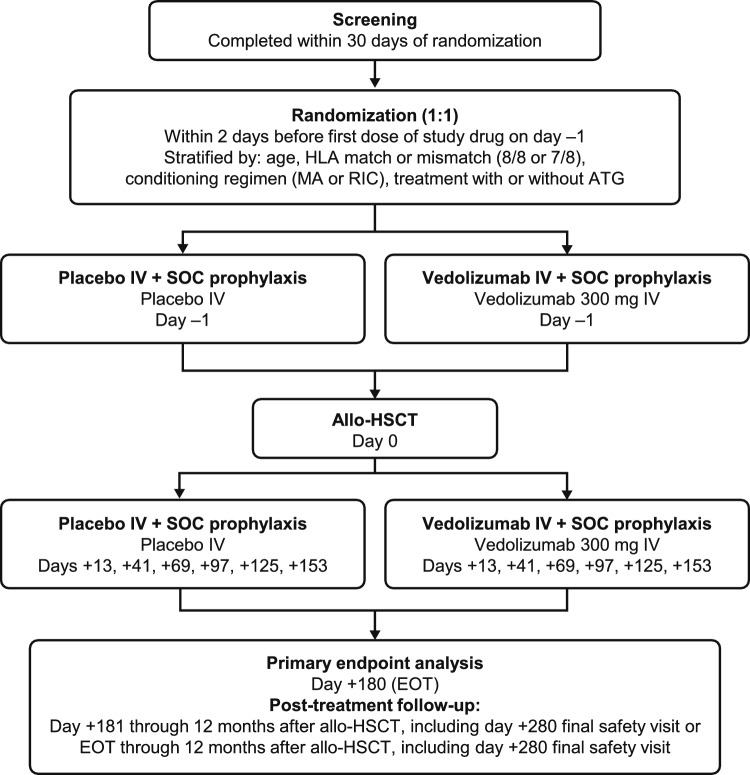
Study design. *EOT* end of treatment, *MA* myeloablative, *SOC* standard of care

The details of the GRAPHITE study have described previously [31]. The study was conducted at 95 sites from February 6, 2019, through to May 9, 2022, including 9 sites in Japan. A protocol amendment allowed for expansion of enrollment at selected sites to include adolescent patients (aged ≥ 12 years). The study consisted of a 30-day pre-transplantation screening period, a 155-day treatment period, an end-of-treatment visit at day 180 after allo-HSCT (or an early-termination visit for patients who discontinued treatment), and a post-treatment follow-up period until up to 1 year after allo-HSCT or until death/study withdrawal or study termination. After screening, patients were randomized at a 1:1 ratio to receive IV vedolizumab (300 mg) or placebo. Patients received 7 IV doses of randomized treatment starting on day –1 before allo-HSCT and on days + 13, + 41, + 69, + 97, + 125, and + 153 after allo-HSCT (Fig. 1). Randomization was stratified by age (patients aged ≥ 18 years or aged 12 to < 18 years); human leukocyte antigen (HLA) match (8/8 or 7/8); conditioning regimen intensity (myeloablative conditioning [MAC] or reduced intensity conditioning [RIC]); and ATG treatment status (with or without ATG). Treatment assignment was carried out using an interactive response technology system within 2 days before the first dose of study treatment. All study site personnel and patients were blinded to treatment assignments during the study, except those directly involved with study medication preparation.

All patients received a standard GVHD prophylaxis regimen consisting of a combination of a calcineurin inhibitor (cyclosporine or tacrolimus) and MTX. At the investigator’s discretion, MMF could be used in place of MTX. Any other agents used for GVHD prophylaxis, apart from corticosteroids and ATG, were prohibited. Other prohibited concomitant medications included live vaccines (within 30 days of randomization to ≥ 6 months after last dose of study treatment), immune checkpoint inhibitors, and any other biologic treatment except for localized injections.

Institutional review boards and independent ethics committees were consulted according to the applicable local requirements, and provided written approval of the protocol and patient informed consent. This study was conducted in compliance with the informed consent regulations as described in the Declaration of Helsinki and the International Conference on Harmonisation Guidelines for Good Clinical Practice, and in accordance with all applicable local laws and regulations. An independent data monitoring committee reviewed the interim analysis results and safety data.

### Patient selection

Patients were eligible if they were ≥ 12 years of age, weighed ≥ 30 kg at randomization, and were candidates for unrelated donor peripheral blood or bone marrow allo-HSCT as treatment for hematologic malignancies or myeloproliferative disorders. After DNA-based matching, eligible patients were 8 of 8 or 7 of 8 HLA matched (a single allele or antigen mismatch at HLA-A, HLA-B, and HLA-C, and HLA-DRB1 was permitted), and met local institutional eligibility criteria for allo-HSCT. In addition, patients had either an Eastern Cooperative Oncology Group Performance Status score of ≤ 2 if they were aged ≥ 18 years, a Karnofsky Performance Status ≥ 60% if they were aged ≥ 16 years, or a Lansky Performance Status ≥ 60% if they were aged 12 to < 16 years (Supplementary Tables S1 and S2) [32]. Patients were excluded if they had received a previous allo-HSCT or had suffered from non-malignant hematological disorders requiring allo-HSCT. A full list of study inclusion and exclusion criteria are provided in Supplementary Table S3.

### Study endpoints and assessments

The primary endpoint was lower-GI aGVHD-free survival by day + 180 after allo-HSCT, defined as the time from the first dose of study treatment (day − 1) to the first clinically diagnosed presentation of any stage lower-GI aGVHD or death from any cause, whichever occurred first. Only cases of lower-GI aGVHD, and not upper-GI aGVHD, or death by day + 180 counted for this analysis (see Supplementary Tables S4–S7 for the aGVHD grading indices used in efficacy endpoints).

Key secondary endpoints analyzed at day + 180 after allo-HSCT included: (1) lower-GI aGVHD-free and relapse-free survival; (2) international bone marrow transplant registry database (IBMTR) Grade C–D aGVHD (any organ)-free survival; (3) non-relapse mortality (NRM); (4) overall survival (OS); and (5) IBMTR Grade B–D aGVHD-free survival. Lower-GI aGVHD-free and relapse-free survival was defined as the time to the first documented Stage 1–4 lower-GI aGVHD, relapse of underlying malignancy, or death from any cause, whichever occurred first. IBMTR Grade C–D aGVHD (any organ)-free survival was defined as the time to the first documented IBMTR Grade C–D aGVHD of any organ or death from any cause, whichever occurred first. NRM was defined as the time from the first dose of study treatment to death, without the occurrence of a relapse. OS was defined as the time from the first dose of study treatment to death from any cause. IBMTR Grade B–D aGVHD-free survival was defined as time to the first documented IBMTR Grade B–D aGVHD of any organ or death from any cause, whichever occurred first. The main exploratory endpoints included the primary and secondary endpoints assessed at day + 365 after allo-HSCT. An additional exploratory endpoint assessed at day + 365 after allo-HSCT was GVHD (any organ)-free and relapse (of the underlying malignancy)-free survival, where an event was defined as death or a Grade 3–4 aGVHD event in any organ (graded according to modified Glucksberg criteria), or chronic GVHD requiring systemic immunosuppression or relapse.

Safety and tolerability endpoints included adverse events (AEs), serious AEs (SAEs), AEs of special interest, vital signs, and laboratory tests (clinical chemistry, hematology, and liver function tests). AEs and SAEs were reported throughout the trial and coded using the Medical Dictionary for Regulatory Activities (version 24.0). Routine safety reviews were conducted by the data monitoring committee.

### Statistical analyses

Statistical analyses were carried out as described previously [31]. Briefly, study enrollment was terminated early, and the study completed on May 9, 2022, primarily due to low patient recruitment and enrollment during the COVID-19 pandemic (from February 2020). The end of study analysis was performed after the final database lock. In this post hoc analysis, all randomized patients receiving ≥ 1 dose of study treatment who underwent allo-HSCT were analyzed for efficacy endpoints and all patients who received ≥ 1 dose of the study treatment were analyzed for safety endpoints. All time-to-event endpoints were analyzed using log-rank tests. Cox proportional hazards models, stratified by randomization stratification factors, were fitted and hazard ratios (HRs) reported. All dichotomous efficacy endpoints were analyzed using Cochran–Mantel–Haenszel tests for risk differences, stratified by randomization stratification factors. To control the overall type I error rate for the comparison between the vedolizumab and placebo groups for the primary and key secondary efficacy endpoints in the final analysis, a fixed-sequence hierarchical testing procedure was used.

## Results

### Patient population

A total of 37 Japanese and 306 non-Japanese patients were randomized. Of these, 1 Japanese patient randomized to vedolizumab and 8 non-Japanese patients (vedolizumab, n = 4; placebo, n = 4) did not receive any study medications and were excluded from the safety analysis. The efficacy analysis consisted of 35 Japanese patients who received ≥ 1 dose of study treatment and allo-HSCT and 298 non-Japanese patients (Supplementary Table S8). Of these, 33 (89.2%) Japanese patients and 249 (81.4%) non-Japanese patients completed the day + 180 study visit. Thirteen (35.1%) Japanese patients discontinued treatment during the study (vedolizumab, n = 8; placebo, n = 5), compared with 134 (43.8%) non-Japanese patients (vedolizumab, n = 58; placebo, n = 76). The reasons for treatment discontinuation are listed in Supplementary Table S8. In addition, 12 (32.4%) Japanese patients (vedolizumab, n = 5; placebo, n = 7) and 116 (37.9%) non-Japanese patients (vedolizumab, n = 52; placebo, n = 64) discontinued the study. Reasons for study discontinuation are listed in Supplementary Table S8. Of the 12 Japanese patients discontinuing the study, the most frequent reasons for study discontinuation were withdrawal by the patient (41.7%; vedolizumab, n = 2; placebo, n = 3) and death (33.3%; vedolizumab, n = 0; placebo, n = 4). The main reasons for study discontinuation in non-Japanese patients were also withdrawal by the patient (25.0%; vedolizumab, n = 14; placebo, n = 15) and death (47.4%; vedolizumab, n = 26; placebo, n = 29). Japanese patients received a mean (standard deviation) 5.5 (2.1) doses of study treatment (vedolizumab, 5.2 [2.2] vs placebo, 5.9 [2.0]); 42.1% received ≥ 7 doses of study treatment in the vedolizumab group and 70.6% in the placebo group. Non-Japanese patients received a mean (standard deviation) 5.2 (2.2) doses of study treatment (vedolizumab, 5.5 [2.0] vs placebo, 5.0 [2.3]); 54.0% and 48.6% received ≥ 7 treatment doses in the vedolizumab and placebo groups, respectively.

Patient demographics and disease characteristics as well as allo-HSCT parameters (for patients included in the efficacy analysis) are summarized in Table 1 for Japanese and non-Japanese patient subgroups. Most baseline patient demographic and disease and transplant characteristics were similar between Japanese and non-Japanese cohorts and were balanced across placebo and vedolizumab treatment groups. In Japanese patients, the median age was 46 years (range 24–70 years), 57.1% were male, and the most frequent primary disease was acute myeloid leukemia (AML) in 40.0% of patients (14/35; vedolizumab, n = 5; placebo, n = 9), followed by myelodysplastic syndrome in 20.0% of patients (7/35; vedolizumab, n = 4; placebo, n = 3) and acute lymphoid leukemia (ALL) also in 20.0% (7/35; vedolizumab, n = 5; placebo, n = 2). Seventeen of 35 Japanese patients (48.6%) had a baseline Eastern Cooperative Oncology Group status score of 1 (vedolizumab, n = 9; placebo, n = 8).

**Table 1:** Patient demographics and disease characteristics and HSCT parameters (full analysis set)

Patient demographics and disease characteristics	Japanese cohort (N = 35)		Non-Japanese cohort (N = 298)	
	PBO (n = 17)	VDZ (n = 18)	PBO (n = 148)	VDZ (n = 150)
Age, years				
Median (range)	49.0 (24–70)	43.5 (31–68)	55.0 (16–74)	55.0 (19–74)
Patients aged > 60 years, n (%)	4 (23.5)	2 (11.1)	54 (36.5)	52 (34.7)
Sex, n (%)				
Male	11 (64.7)	9 (50.0)	95 (64.2)	94 (62.7)
Female	6 (35.3)	9 (50.0)	53 (35.8)	56 (37.3)
Primary disease, n (%)				
AML	9 (52.9)	5 (27.8)	63 (42.6)	68 (45.3)
ALL	2 (11.8)	5 (27.8)	22 (14.9)	24 (16.0)
MDS	3 (17.6)	4 (22.2)	33 (22.3)	30 (20.0)
Other myeloproliferative	1 (5.9)	2 (11.1)	13 (8.8)	14 (9.3)
CML	0	1 (5.6)	5 (3.4)	5 (3.3)
CLL	0	0	0	1 (0.7)
Non-Hodgkin lymphoma	2 (11.8)	1 (5.6)	12 (8.1)	6 (4.0)
Hodgkin lymphoma	0	0	0	2 (1.3)
ECOG^a^ status, n (%)				
0	5 (29.4)	3 (16.7)	60 (40.8)	61 (40.7)
1	8 (47.1)	9 (50.0)	78 (53.1)	80 (53.3)
2	4 (23.5)	6 (33.3)	9 (6.1)	8 (5.3)
3	0	0	0	1 (0.7)
HSCT parameter, n (%)				
Stem cell source				
Bone marrow	14 (82.4)	13 (72.2)	8 (5.4)	14 (9.3)
Peripheral blood	3 (17.6)	5 (27.8)	139 (94.6)	136 (90.7)
HLA compatability				
7/8 matched	4 (23.5)	4 (22.2)	11 (7.4)	12 (8.0)
8/8 matched	13 (76.5)	14 (77.8)	137 (92.6)	138 (92.0)
Disease status (AML and ALL)				
Complete remission 1	10 (90.9)	5 (50)	69 (83.1)	65 (73.9)
Complete remission > 1	1 (9.1)	5 (50)	14 (16.9)	21 (23.9)
Conditioning regimen^b^				
Myeloablative	13 (76.5)	13 (72.2)	76 (51.4)	75 (50.0)
Recued intensity	4 (23.5)	5 (27.8)	72 (48.6)	75 (50.0)
ATG^b^				
With	2 (11.8)	4 (22.2)	64 (43.2)	67 (44.7)
Without	15 (88.2)	14 (77.8)	84 (56.8)	83 (55.3)

Full analysis set: all patients who received ≥ 1 dose of study treatment and received allo-HSCT; of 334 patients who received ≥ 1 dose of study treatment, 333 also received allo-HSCT (full analysis set)

*Allo-HSCT* allogenic hematopoietic stem cell transplantation therapy, *ALL* acute lymphoid leukemia, *AML* acute myeloid leukemia, *ATG* anti-thymocyte globulin, *CLL* chronic lymphocytic leukemia, *CML* chronic myeloid leukemia, *ECOG* Eastern Cooperative Oncology Group, *HLA* human leukocyte antigen, *HSCT* hematopoietic stem cell transplantation, *MDS* myelodysplastic syndrome, *PBO* placebo, *VDZ* vedolizumab

^a^ECOG no patients had ECOG 4 status

^b^Data collected at randomization

There were some differences in baseline characteristics between the Japanese and non-Japanese cohorts. More Japanese patients than non-Japanese patients were aged < 40 years (34.3% vs 23.5%) or 40–60 years (48.6% vs 40.9%), whereas fewer were aged ≥ 60 years (17.1% vs 35.6%). A lower proportion of Japanese (17.1%; 6/35; vedolizumab, n = 4; placebo, n = 2) than non-Japanese patients (44.0%; 131/298; vedolizumab, n = 67; placebo, n = 64) received ATG as part of GVHD prophylaxis. Japanese patients preferentially received MAC (74.3%; 26/35; vedolizumab, n = 13; placebo, n = 13) than RIC (25.7%; 9/35; vedolizumab, n = 5; placebo, n = 4), whereas the proportion of non-Japanese patients who received MAC (50.7%; 151/298; vedolizumab, n = 75; placebo, n = 76) was similar to the proportion who received RIC (49.3%; 147/298; vedolizumab, n = 75; placebo, n = 72). The majority of Japanese patients received bone marrow grafts (77.1%; 27/35; vedolizumab, n = 13; placebo, n = 14) whereas a low proportion of non-Japanese patients received bone marrow (7.4%; 22/298; vedolizumab, n = 14; placebo, n = 8). The reverse was true for peripheral blood grafts, with 22.9% (8/35; vedolizumab, n = 5; placebo, n = 3) of Japanese patients and 92.6% (275/298; vedolizumab, n = 136; placebo, n = 139) of non-Japanese patients receiving peripheral blood grafts. HLA 1-locus mismatched donors were used in a greater proportion of Japanese allo-HSCT recipients (22.9%; 8/35) than non-Japanese allo-HSCT recipients (7.7%; 23/298). HLA 1-locus mismatched donors were used in a similar proportion of patients who received vedolizumab versus placebo, in both Japanese (22.2% [4/18] for vedolizumab vs 23.5% [4/17] for placebo) and non-Japanese (8.0% [12/150] for vedolizumab vs 7.4% [11/148] for placebo) subgroups.

### Engraftment

In Japanese patients, neutrophil engraftment occurred in 18 of 19 patients (94.7%) in the vedolizumab treatment group and 16 of 17 patients (94.1%) in the placebo group. The median (range) time to neutrophil engraftment was 17.5 (13–24) days in the vedolizumab group and 18.0 (14–26) days in the placebo group. Platelet engraftment occurred in 18 of 19 (94.7%) patients in the vedolizumab group and 15 of 17 (88.2%) patients in the placebo group. The median (range) time to platelet engraftment was 30.0 (20–104) days in the vedolizumab group and 29.0 (21–233) days in the placebo group.

In non-Japanese patients, neutrophil engraftment occurred in 147 of 150 patients (98.0%) in the vedolizumab group and 144 of 148 patients (97.3%) in the placebo group. The median (range) time to neutrophil engraftment was 16.0 (8–35) and 15.0 (8–31) days in the vedolizumab and placebo groups, respectively. Platelet engraftment occurred in 141 of 150 patients (94.0%) in the vedolizumab group and 133 of 148 (89.9%) in the placebo group. The median (range) time to platelet engraftment was 17.0 (1–136) and 16.0 (0–152) days in these groups, respectively.

### Efficacy endpoints

**Fig. 2:**
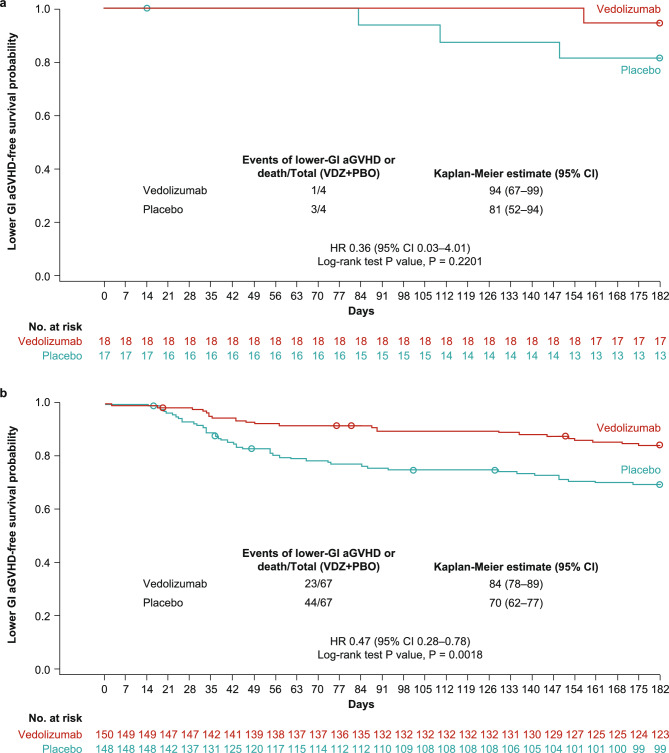
Kaplan-Meier curves for lower-GI aGVHD survival by day +180 after allo-HSCT in **a** Japanese and **b** non-Japanese patients. *PBO* placebo, *VDZ* vedolizumab

**Fig. 3:**
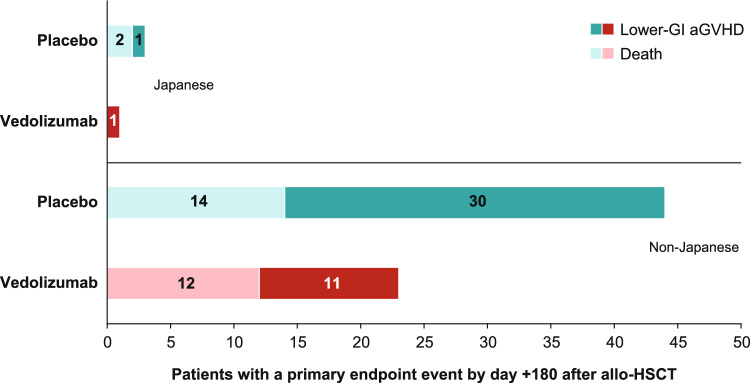
Patients with a primary endpoint event of lower-GI aGVHD or death by day +180 after allo-HSCT. Data are from the full analysis set, which included all patients in the Japanese or non-Japanese cohorts who received at least 1 dose of study treatment and received allo-HSCT. Graph shows the number of patients with a lower-GI aGVHD event or death event for the primary endpoint analysis of lower-GI aGVHD-free survival at day +180 after allo-HSCT

In Japanese patients, the Kaplan–Meier estimate of the primary endpoint (lower-GI aGVHD-free survival by day + 180) was numerically higher in the vedolizumab than the placebo group (vedolizumab, 94% [95% confidence interval (CI) 67–99]; placebo, 81% [95% CI 52–94]; HR 0.36 [95% CI 0.03–4.01]; P = 0.2201) (Fig. 2a). Overall, for the primary study endpoint assessed in non-Japanese patients, lower-GI aGVHD-free survival rates by day + 180 were numerically lower compared with Japanese patients, and these rates were significantly higher in non-Japanese patients who received vedolizumab versus placebo (vedolizumab, 84% [95% CI 78–89]; placebo, 70% [95% CI 62–77]; HR 0.47 [95% CI 0.28–0.78]; P = 0.0018) (Fig. 2b). At day + 180 after allo-HSCT, events of lower-GI aGVHD or death were reported in 1 Japanese patient in the vedolizumab group (5.6%) versus 3 in the placebo group (17.6%), and 23 non-Japanese patients in the vedolizumab group (15.3%) versus 44 in the placebo group (29.7%) (Fig. 3). For the frequency of lower-GI aGVHD by maximum clinical stage (Supplementary Table S9), there was 1 case of Stage 1 lower-GI aGVHD in the vedolizumab group and 1 case of Stage 2 lower-GI aGVHD in the placebo group of the Japanese cohort by day + 180 after allo-HSCT; frequencies of Stage 2–4 aGVHD of the skin and liver were similar between treatment groups at this time point. For non-Japanese patients, there were 4 cases of Stage 2–4 lower-GI aGVHD in the vedolizumab group compared with 13 cases in the placebo group by day + 180 after allo-HSCT; the frequencies of Stage 2–4 aGVHD of the skin and liver were similar between treatment groups.

The Kaplan–Meier curves for the key secondary endpoints analyzed at day + 180 after allo-HSCT in Japanese patients are shown in Supplementary Figure S1. Events of lower-GI aGVHD and relapse of the underlying malignancy occurred in 2 (11.1%) patients in the vedolizumab group and 3 (17.6%) in the placebo group (HR 0.76 [95% CI 0.10–5.47]; P = 0.4986). Events of IBMTR Grade C–D aGVHD of any organ occurred in 2 (11.1%) patients in the vedolizumab group and 5 (29.4%) in the placebo group (HR 0.36 [95% CI 0.07–2.03]; P = 0.1167). No NRM events were reported for patients in the vedolizumab group by the day + 180 time point but occurred in 2 (11.8%) patients in the placebo group (P = 0.1274). OS events did not occur in patients from the vedolizumab group but were reported for 2 (11.8%) patients in the placebo group (P = 0.1274). Events of IBMTR Grade B–D aGVHD of any organ occurred in 4 (22.2%) patients from the vedolizumab group and 6 (35.3%) in the placebo group (HR 0.68 [95% CI 0.18–2.98]; P = 0.3002).

The Kaplan–Meier curves for the key secondary endpoints analyzed at day + 180 after allo-HSCT in non-Japanese patients are shown in Supplementary Figure S2. Rates of lower-GI aGVHD-free and relapse of the underlying malignancy–free survival by day + 180 were significantly higher in vedolizumab-treated patients compared with those receiving placebo. Events of lower-GI aGVHD and relapse of the underlying malignancy were reported in 33 (22.0%) patients in the vedolizumab group and 53 (35.8%) in the placebo group (HR 0.57 [95% CI 0.37–0.89]; P = 0.0057). IBMTR Grade C–D aGVHD-free survival by day + 180 was significantly higher in vedolizumab-treated patients compared with those receiving placebo. Events of IBMTR Grade C–D aGVHD of any organ and relapse occurred in 33 (22.0%) patients in the vedolizumab group and 47 (31.8%) in the placebo group (HR 0.64 [95% CI 0.41–0.99]; P = 0.050). NRM events occurred in 10 (6.7%) patients in the vedolizumab group and 17 (11.5%) in the placebo group (HR 0.56 [95% CI 0.26–1.23]; P = 0.1415). All-cause mortality events captured for the endpoint of OS occurred in 17 (11.3%) patients in the vedolizumab group and 23 (15.5%) in the placebo group (HR 0.71 [95% CI 0.38–1.33]; P = 0.2615). Events of IBMTR Grade B–D aGVHD of any organ were reported in 52 (34.7%) patients in the vedolizumab group and 71 (48.0%) in the placebo treatment group (HR 0.67 [95% CI 0.46–0.96]; P = 0.0190) (Table 2).

**Table 2:** Key secondary endpoint results in Japanese and non-Japanese patients

Key secondary endpoints (by day + 180)	Japanese				Non-Japanese			
	PBO (n = 17)	VDZ (n = 18)	VDZ vs PBO		PBO (n = 148)	VDZ (n = 150)	VDZ vs PBO	
	Events, n (%)		P value^a^	HR (95% CI)^b^	Events, n (%)		P value^a^	HR (95% CI)^b^
1. Lower Gl aGVHD-free and relapse-free (of the underlying malignancy) survival								
Events of lower-GI aGVHD, relapse or death	3 (17.6)	2 (11.1)	0.4986	0.76 (0.10–5.47)	53 (35.8)	33 (22.0)	0.0057*	0.57 (0.37–0.89)
2. IBMTR^c^ Grade C–D aGVHD-free (any organ involvement) survival								
Events of Grade C–D aGVHD or death	5 (29.4)	2 (11.1)	0.1167	0.36 (0.07–2.03)	47 (31.8)	33 (22.0)	0.0500*	0.64 (0.41–0.99)
3. Non-relapse mortality	2 (11.8)	0	0.1274	0 (0 to NE)	17 (11.5)	10 (6.7)	0.1415	0.56 (0.26–1.23)
4. Overall survival	2 (11.8)	0	0.1274	0 (0 to NE)	23 (15.5)	17 (11.3)	0.2615	0.71 (0.38–1.33)
5. IBMTR^c^ Grade B–D aGVHD-free (any organ involvement) survival								
Events of Grade B–D aGVHD or death	6 (35.3)	4 (22.2)	0.3002	0.68 (0.18–2.58)	71 (48.0)	52 (34.7)	0.0190	0.67 (0.46–0.96)

*aGVHD* acute graft-versus-host disease, *CI* confidence interval, *GI* gastrointestinal, *HR* hazard ratio, *IBMTR* International Bone Marrow Transplant Register, *PBO* placebo, *VDZ* vedolizumab

^*^Statistically significant for full analysis set: all patients who received ≥ 1 dose of study treatment and received allogenic hematopoietic stem cell transplantation. Primary and 5 key secondary efficacy endpoints were tested following a fixed-sequence hierarchical testing procedure. When 1 efficacy endpoint was found not significant (i.e., P > 0.05), testing of all subsequent endpoints would not be performed

^a^P value was obtained from log-rank tests

^b^HRs and 95% CIs were obtained from Cox proportional hazards models with treatment group stratified by randomization strata: human leukocyte antigen match (7/8, 8/8), conditioning regimen (myeloablative conditioning, reduced intensity conditioning), and anti-thymocyte globulin (with, without)

^c^IBMTR Severity Index for aGVHD Grade C–D is equivalent to maximum Stage 3–4 skin or liver or GI tract, Grade B–D is maximum Stage 2–4 skin or Stage 1–2 to 4 liver or Stage 1–2 to 4 GI tract (adapted from Rowlings et al. *Br J Haematol* 1997;97:855–64)

The results of the exploratory endpoints, analyzed at day + 365 after allo-HSCT, were consistent with the results of the primary and secondary efficacy endpoints in both Japanese and non-Japanese patients (Supplementary Table S10). By day + 365 after allo-HSCT, 2 (11.1%) Japanese patients in the vedolizumab group and 4 (23.5%) in the placebo group had an event of lower-GI aGVHD or death (HR 0.53 [95% CI 0.09–3.22]; nominal P = 0.2844). In non-Japanese patients, lower-GI aGVHD-free survival by day + 365 after allo-HSCT was higher in vedolizumab-treated patients compared with those receiving placebo; 34 (22.7%) patients in the vedolizumab group had an event of lower-GI aGVHD or death by day + 365 compared with 52 (35.1%) in the placebo group (HR 0.55 [95% CI 0.36–0.86]; nominal P = 0.0075). GVHD (aGVHD Grade 3–4 modified Glucksberg and chronic GVHD requiring systemic immunosuppression) and relapse (of the underlying malignancy)-free survival events in Japanese patients by day + 365 after allo-HSCT were reported in 3 (16.7%) patients in the vedolizumab group and 5 (29.4%) patients in the placebo group (HR 0.61 [95% CI 0.14–2.76]; nominal P = 0.2967). In non-Japanese patients, these events occurred in 53 (35.3%) patients in the vedolizumab group and 59 (39.9%) in the placebo group (HR 0.83 [95% CI 0.57–1.21]; nominal P = 0.2616). For progression-free survival in Japanese patients by day + 365 after allo-HSCT, events of relapse of the underlying malignancy or death occurred in 3 (16.7%) patients in the vedolizumab group and 4 (23.5%) in the placebo group (HR 0.83 [95% CI 0.17–4.17]; nominal P = 0.5564). Relapse events counted for this endpoint occurred in 2 (11.1%) patients in the vedolizumab group and 1 (5.9%) in the placebo group. In non-Japanese patients, events of relapse and death counted for the progression-free survival endpoint occurred in 45 (30.0%) patients in the vedolizumab group and 43 (29.1%) in the placebo group (HR 1.01 [95% CI 0.67–1.55]; nominal P = 0.9971). Relapse events occurred in 31 (20.7) and 21 (14.2%) patients treated with vedolizumab and placebo, respectively.

### Safety

Safety analyses included 36 Japanese patients (vedolizumab, n = 19; placebo, n = 17) and 298 non-Japanese patients (vedolizumab, n = 150; placebo, n = 148). The median (range) treatment exposures were similar between Japanese (vedolizumab, 278.0 [127–283] days; placebo, 280 [127–296] days) and non-Japanese (vedolizumab, 280.0 [127–295] days; placebo, 266 [127–287] days) cohorts.

In the Japanese cohort, AEs of Grade 3 or higher occurred in 94.7% of patients who received vedolizumab (treatment related in 5 [26.3%]) and 94.1% who received placebo (treatment related in 3 [17.6%]). The most frequent treatment-related AEs by type were infections (n = 3) for the vedolizumab treatment group and immune system disorders (n = 3), GI disorders (n = 2), and infections (n = 2) for the placebo group. AEs led to treatment discontinuation in 6 (31.6%) and 5 (29.4%) patients in these groups, respectively (Table 3). SAEs occurred in 11 (57.9%) patients who received vedolizumab and 9 (52.9%) who received placebo. Of these, 3 patients had 3 SAEs in the vedolizumab group that were considered as treatment related by the study investigator (pancytopenia, pyrexia, and bronchopulmonary aspergillosis), and 2 patients had 3 SAEs in the placebo group considered as treatment related (atypical mycobacterial infection, pneumonia, and increased alanine aminotransferase). One patient (5.3%) died in the vedolizumab group (aGVHD in the liver) and 3 (17.6%) patients died in the placebo group (interstitial lung disease, myelodysplastic syndrome, and pneumonia). None of the deaths were causally related to the study treatment.

**Table 3:** Adverse events

Patients, n (%)	Japanese		Non-Japanese	
	PBO (n = 17)	VDZ (n = 19)	PBO (n = 148)	VDZ (n = 150)
Any AEs^a^	17 (100)	19 (100)	148 (100)	150 (100)
Grade ≥ 3	16 (94.1)	18 (94.7)	131 (88.5)	138 (92.0)
Grade ≥ 3 study treatment-related^b^	3 (17.6)	5 (26.3)	16 (10.8)	13 (8.7)
Leading to study treatment discontinuation	5 (29.4)	6 (31.6)	46 (31.1)	38 (25.3)
SAE	9 (52.9)	11 (57.9)	105 (70.9)	109 (72.7)
Study treatment related	2 (11.8)	3 (15.8)	12 (8.1)	8 (5.3)
Leading to study treatment discontinuation	4 (23.5)	5 (26.3)	34 (23.0)	34 (22.7)
AESI				
Serious infections^c^	15 (88.2)	16 (84.2)	96 (64.9)	109 (72.7)
PML^d^	0	0	2 (1.4)	3 (2.0)
Malignancy^e^	1 (5.9)	1 (5.3)	25 (16.9)	28 (18.7)
Liver injury	11 (64.7)	10 (52.6)	58 (39.2)	58 (38.7)
Hypersensitivity/injection site reactions	16 (94.1)	16 (84.2)	120 (81.1)	118 (78.7)
Leukopenia/lymphopenia	1 (5.9)	0	6 (4.1)	14 (9.3)
Reactivation of CMV	8 (47.1)	6 (31.6)	30 (20.3)	39 (26.0)
Deaths	3 (17.6)	1 (5.3)	24 (16.2)	20 (13.3)

Medical Dictionary for Regulatory Activities (version 24.0) was used for coding AEs

*AE* adverse event, *AESI* adverse event of special interest, *CMV* cytomegalovirus, *PBO* placebo, *PML* progressive multifocal leukoencephalopathy, *SAE* serious adverse event, *VDZ* vedolizumab

^a^AE/SAE is defined as any AE/SAE newly occurring or worsening from the first dose and 18 weeks after last dose of study treatment

^b^Defined as AEs/SAEs that, in the study investigator's opinion, were related to administration of study treatment (VDZ or PBO)

^c^Defined as any serious infection or infestation event not classified as a preferred term of CMV colitis

^d^Suspected PML cases were 5 patients with an AE of human polyomavirus infection; none of these were diagnosed as PML. A single PML case was confirmed after the last safety follow-up and was considered unrelated to study treatment

^e^All cases of malignancies including relapse of primary disease

In the non-Japanese cohort, AEs of Grade 3 or higher occurred in 92.0% of patients who received vedolizumab (treatment related in 13 [8.7%]) and 88.5% who received placebo (treatment related in 16 [10.8%]). The most frequent treatment-related AEs by type were from investigations (n = 9), infections (n = 8), blood and lymphatic disorders (n = 5), and skin and subcutaneous tissue disorders (n = 5) for the vedolizumab group and investigations (n = 12), GI disorders (n = 9), and immune system disorders (n = 6) for the placebo group. AEs led to treatment discontinuation in 38 (25.3%) and 46 (31.1%) patients in these treatment groups, respectively (Table 3). Serious AEs occurred in 109 (72.7%) patients who received vedolizumab and 105 (70.9%) who received placebo. Eight patients had 10 SAEs in the vedolizumab group considered as treatment related by the study investigator (n = 1 patient each for ventricular tachycardia, aGVHD of the intestine, chronic GVHD, *Escherichia* bacteremia, pneumonia, pseudomonal pneumonia, viral meningitis, subdural hematoma, increased alanine aminotransferase, and recurrent chronic myeloid leukemia). Twelve patients had 12 SAEs in the placebo group that were considered treatment related (abnormal hepatic function [n = 2]; n = 1 patient each for myocardial infarction, diarrhea, aGVHD of the intestine, aGVHD of the liver, encephalitis, rhinovirus infection, sepsis, abnormal alanine aminotransferase, abnormal liver function test, and macular rash). There were 44 non-Japanese patients who died during the study; 20 (13.3%) in the vedolizumab group and 24 (16.2%) in the placebo group. No deaths were causally related to the study treatment.

For AEs of special interest in the Japanese cohort, ≥ 1 serious infection occurred in 16 (84.2%) patients receiving vedolizumab and 15 (88.2%) patients receiving placebo. In the non-Japanese cohort, ≥ 1 serious infection occurred in 109 (72.7%) patients receiving vedolizumab and 96 (64.9%) receiving placebo. No cases of PML occurred in Japanese patients whereas 5 cases of suspected PML occurred in non-Japanese patients (vedolizumab, n = 3; placebo, n = 2) with an AE of human polyomavirus infection; none of these were diagnosed as PML. A single PML case was confirmed after the last safety follow-up and was considered unrelated to study treatment. No cases of CMV colitis occurred in Japanese patients whereas 2 cases of CMV colitis occurred in non-Japanese patients (1 patient in each treatment group). For AEs that included the term “colitis,” incidence rates were low in both Japanese and non-Japanese patients (Supplementary Table S11). Post-transplant lymphoproliferative disorders were not reported in the Japanese patient cohort during the study, whereas 3 non-Japanese patients had post-transplant lymphoproliferative disorders (all placebo treated).

## Discussion

This post hoc analysis of the GRAPHITE phase 3, randomized, double-blind trial investigated the efficacy and safety of vedolizumab versus placebo for the prevention of lower-GI aGVHD when added to standard aGVHD prophylaxis (cyclosporine or tacrolimus and MTX or MMF + / − ATG). Data from 35 Japanese unrelated allo-HSCT recipients were analyzed. Although the Japanese cohort size was small and not powered to show differences between treatments, the results showed that rates of lower-GI aGVHD-free survival at day + 180 after allo-HSCT (the primary endpoint) were numerically higher in vedolizumab-treated patients than those receiving placebo. The outcomes for each of the efficacy endpoints analyzed in Japanese patients appeared consistently more favorable for vedolizumab-treated patients compared with those in the placebo group. In the larger non-Japanese cohort, lower-GI aGVHD-free survival at day + 180 and day + 365 after allo-HSCT were significantly higher for the vedolizumab versus placebo groups. Secondary endpoints of lower-GI aGVHD-free and relapse-free survival, and Grade C–D aGVHD-free survival, were also significantly in favor of vedolizumab over placebo.

Our results are consistent with those from the overall GRAPHITE study population [31]. In the overall study, the primary endpoint was met, showing that vedolizumab was more effective than placebo for the prevention of lower-GI aGVHD. Rates of lower-GI aGVHD-free survival by day + 180 after allo-HSCT were 85.5% and 70.9% for vedolizumab and placebo treatment respectively (HR 0.45 [95% CI 0.27–0.73]; P < 0.001). Secondary endpoints of lower-GI aGVHD-free and relapse-free survival (HR 0.56 [0.37–0.86]; P = 0.0043), and Grade C–D aGVHD-free survival (HR 0.59 [0.39–0.91]; P = 0.0204) were also significantly in favor of vedolizumab treatment over placebo.

There were no differences between placebo and vedolizumab treatment groups in safety endpoints for Japanese patients, and the proportion of patients with SAEs and serious infections were similar between treatment groups. Although there were more deaths in the placebo group than the vedolizumab group, no deaths were treatment related. Safety parameters analyzed in non-Japanese patients also showed no obvious treatment-related differences. The proportion of patients with aGVHD of the skin and liver were comparable between the placebo and vedolizumab groups, but whether vedolizumab can reduce the severity of extraintestinal aGVHD requires further investigation. No new safety concerns were identified for vedolizumab (IV 300 mg) when used as aGVHD prophylaxis in Japanese patients in this study. This finding is consistent with vedolizumab safety data obtained from clinical trials of Japanese patients with inflammatory bowel disease [33, 34].

The main limitation of this analysis in Japanese patients was the relatively small number of patients included, and that this subgroup was not powered to show statistical differences between treatments. There were also some differences in the background transplant characteristics between Japanese and non-Japanese HSCT patients, such as a higher proportion with bone marrow stem cell source HSCT in Japanese patients compared with a higher proportion with peripheral blood HSCT in non-Japanese patients, a slightly higher proportion of HLA 7/8 matched donors in the Japanese cohort, more Japanese patients using MAC conditioning regimens, and a slightly lower rate of ATG use in Japanese patients.

The efficacy of GI-GVHD prophylaxis in cord blood transplantation, which is widely performed in Japan, has not been verified. In addition, assessment of the treatment effect of vedolizumab in the setting of haploidentical hematopoietic cell transplantation is also warranted, especially focusing on the combinational use of vedolizumab with post-transplant cyclophosphamide.

In conclusion, vedolizumab in combination with standard calcineurin inhibitor–based GVHD prophylaxis demonstrated a trend for a higher probability of lower-GI aGVHD-free survival, without additional safety concerns, in the subgroup of Japanese patients. However, a statistically significant difference was not observed due to the small sample size in this subgroup analysis; thus, accumulation of post-marketing real-world data is needed.

## Supplementary Information

Below is the link to the electronic supplementary material.

Supplementary file1 (DOCX 7003 KB)

## Data Availability

The datasets, including the redacted study protocol, redacted statistical analysis plan, and individual participants’ data supporting the results reported in this article, will be made available within 3 months from initial request to researchers who provide a methodologically sound proposal. The data will be provided after its de-identification, in compliance with applicable privacy laws, data protection, and requirements for consent and anonymization.
