# Supplementary material for: Vedolizumab for prevention of lower-GI acute GVHD in the Japanese subgroup analysis of the phase 3 GRAPHITE study
Source: Int J Hematol. 2025 Mar 12;122(1):93–105. doi: 10.1007/s12185-025-03955-9 (PMC12202567; doi:10.1007/s12185-025-03955-9)

# Supplementary Information

**Supplementary Table S1** ECOG Performance Status Scale (for patients aged ≥18 years) [1]

| Grade | Description |
| --- | --- |
| 0 | Normal activity. Fully active, able to carry on all pre-disease performance without restriction. |
| 1 | Symptoms but ambulatory. Restricted in physically strenuous activity but ambulatory and able to carry out work of a light or sedentary nature (e.g., light housework, office work). |
| 2 | In bed <50% of the time. Ambulatory and capable of all self-care but unable to carry out any work activities. Up and about >50% of waking hours. |
| 3 | In bed >50% of the time. Capable of only limited self-care, confined to bed or chair >50% of waking hours. |
| 4 | 100% bedridden. Completely disabled. Cannot carry on any self-care. Totally confined to bed or chair. |
| 5 | Dead. |

*ECOG* Eastern Cooperative Oncology Group.

1. Oken MM, Creech RH, Tormey DC, Horton J, Davis TE, McFadden ET, Carbone PP. Toxicity and response criteria of the Eastern Cooperative Oncology Group. Am J Clin Oncol. 1982;5(6):649–55.

**Supplementary Table S2** Karnofsky and Lansky Performance Status Scales (for patients aged ≥16 and 12 to <16 years, respectively) [1]

| Karnofsky Scale (recipient age ≥16 years) | | Lansky Scale (recipient age 12 to <16 years) | | |
| --- | --- | --- | --- | --- |
| Score | Description | Score | | Description |
| Able to carry on normal activity; no special care is needed | | Able to carry on normal activity; no special care is needed | | |
| 100 | Normal, no complaints, no evidence of disease | 100 | Fully active | |
| 90 | Able to carry on normal activity | 90 | Minor restriction in physically strenuous play | |
| 80 | Normal activity with effort | 80 | Restricted in strenuous play, tires more easily, otherwise active | |
| Unable to work, able to live at home, cares for most personal needs, a varying amount of assistance is needed | | Mild to moderate restriction | | |
| 70 | Cares for self, unable to carry on normal activity or to do active work | 70 | Both greater restrictions of, and less time spent in active play | |
| 60 | Requires occasional assistance but is able to care for most needs | 60 | Ambulatory up to 50% of time, limited active play with assistance/supervision | |
| 50 | Requires considerable assistance and frequent medical care | 50 | Considerable assistance required for any active play, fully able to engage in quiet play | |
| Unable to care for self, requires equivalent of institutional or hospital care, disease may be progressing rapidly | | Moderate to severe restriction | | |
| 40 | Disabled, requires special care and assistance | 40 | Able to initiate quiet activities | |
| 30 | Severely disabled, hospitalization indicated, although death not imminent | 30 | Needs considerable assistance for quiet activity | |
| 20 | Very sick, hospitalization necessary | 20 | Limited to very passive activity initiated by others (e.g., TV) | |
| 10 | Moribund, fatal process progressing rapidly | 10 | Completely disabled, not even passive play | |
| 0 | Dead |  |  | |

1. Center for International Blood & Marrow Transplant Research. Forms Instruction Manual. Appendix L: Karnofsky/Lansky Performance Status. 2009. <https://cdn.manula.com/user/3235/3235_3360_3509_en_1418684645.pdf?v=20230828103227>.

**Supplementary Table S3** Patient eligibility criteria

| Inclusion criteria | |
| --- | --- |
| 1 | The patient or, when applicable, the patient’s legally acceptable representative voluntarily signs and dates a written, informed consent form and any required privacy authorization before performance of any study-related procedures not part of standard medical care, with the understanding that consent may be withdrawn by the patient at any time without prejudice to future medical care. As applicable, a parent/both parents or legally acceptable representative must provide signature of informed consent, and there must be documentation of age-appropriate assent by the patient. |
| 2 | Male or female patients ≥18 years of age and, in selected countries (where local requirements permit and based on feasibility), adolescents aged ≥12 years and weighing ≥30 kg at time of randomization. |
| 3 | Patients must undergo DNA-based HLA matching and be 8 of 8 or 7 of 8 HLA-matched (single allele or antigen mismatch at HLA-A, HLA-B, and HLA-C, and HLA-DRB1 is allowable) unrelated HSCT from either peripheral blood or bone marrow stem cells for a hematologic malignancy or myeloproliferative disorder. Patients should follow local practice for additional HLA match (e.g., France, 9/10 or 10/10 HLA match). |
| 4 | Patients for whom a myeloablative conditioning or RIC is planned. |
| 5 | Allo-HSCT eligible (meeting institutional criteria)-patients planned medical care should include aGVHD prophylaxis with a combination of CNI (CYS or TAC) and MTX or CNI and MMF. With the exception of ATG (ATG-F or thymoglobulin), all other therapies, approved or investigational, for GVHD prophylaxis are excluded. |
| 6 | Status of the primary disease as follows: a) Patients with acute leukemia or chronic myelogenous leukemia: no circulating blasts and <5% blasts in the bone marrow. b) Patients with myelodysplasia: no circulating blasts and <10% blasts in the bone marrow. c) Patients with chronic lymphocytic leukemia or small lymphocytic lymphoma with chemosensitive disease at the time of transplantation (partial or complete response to last salvage therapy). d) Patients with other non-Hodgkin or Hodgkin lymphoma with a response to last salvage therapy or chemosensitive disease per institutional standards at the time of transplantation. e) For patients with myelofibrosis and other myeloproliferative disorders: <5% blasts in the blood and bone marrow. |
| 7 | ECOG [1] Performance Status score of ≤2 for patients aged ≥18 years at randomization, ≥60% using the Karnofsky Performance Status for adolescent patients aged ≥16 years at randomization, or the Lansky Performance Status for adolescent patients aged 12 to <16 years at randomization [2]. |
| 8 | Sufficient cognitive ability to reliably complete the PML checklist at baseline. |
| 9 | Female patients who are:  Postmenopausal for ≥1 year before signing of the informed consent, OR surgically sterile, OR   - If they are aged ≥12 years and not postmenopausal or surgically sterilized must use a highly effective method of contraception during the study and through 18 weeks after the last dose of study drug, OR - Agree to practice true abstinence, when this is in line with the preferred and usual lifestyle of the patient.   Male patients, even if surgically sterilized (i.e., status post vasectomy), who:   - Agree to practice an acceptable effective barrier method of contraception during the entire study treatment period and through 18 weeks after the last dose of study drug, OR - Agree to practice true abstinence, when this is in line with the preferred and usual lifestyle of the patient. |
| 10 | Suitable venous access for the study-required blood sampling, including PK sampling. |
| Exclusion criteria | |
| 1 | Prior allo-HSCT. |
| 2 | Planned umbilical cord blood transplant or planned to receive post-transplant cyclophosphamide, in vivo or ex vivo T cell–depleted HSCs, with the exception of ATG (ATG-F or thymoglobulin). |
| 3 | Planned allo-HSCT for non-malignant hematologic disorders (e.g., aplastic anemia, sickle cell anemia, thalassemia, Fanconi anemia, or immunodeficiency). |
| 4 | Known active cerebral/meningeal disease (including central nervous system involvement of the primary disease), or signs or symptoms of PML, any history of PML, or a positive PML subjective checklist before the administration of study medication on day −1. |
| 5 | Evidence of encephalopathy at screening. |
| 6 | History of any major neurological disorder, including stroke, multiple sclerosis, brain tumor, or neurodegenerative disease. |
| 7 | Prior or current therapy with α4 and/or β7 integrin inhibitors (including, but not limited to natalizumab, etrolizumab, AMG-181), MAdCAM-1-antibodies, anti-CD11a mAb (e.g., efalizumab) within 60 days or 5 half-lives, whichever is longer from randomization. |
| 8 | Prior known exposure of the transplant recipient to vedolizumab. |
| 9 | Any serious medical or psychiatric condition that could, in the investigator’s or medical monitor’s opinion, potentially interfere with the completion of treatment according to this protocol. |
| 10 | Any unstable or uncontrolled cardiovascular, pulmonary, hepatic, renal, GI, genitourinary, coagulation, immunological, endocrine/metabolic, neurologic, or other medical disorder not related to the patient’s primary disease that, in the opinion of the investigator, would confound the study results or compromise patient safety. |
| 11 | Clinically active systemic infection during screening. |
| 12 | Clinically active CMV colitis during screening. |
| 13 | Clinically active *Clostridium difficile* infection or other intestinal pathogen during screening. |
| 14 | Active or latent tuberculosis, regardless of treatment history, as evidenced by any of the following: history of tuberculosis, OR positive QuantiFERON test or T-spot or 2 successive indeterminate QuantiFERON or T-spot tests, OR a tuberculin skin test reaction ≥10 mm (≥5 mm in patients receiving the equivalent of >15 mg/day prednisone). |
| 15 | Chronic hepatitis B (hepatitis B surface antigen positive) or hepatitis C infection (evident by active viral replication by polymerase chain reaction if hepatitis C virus antibody positive). Hepatitis B core antibody positive and negative for hepatitis B surface antigen may be enrolled if viral DNA is undetectable. |
| 16 | History of human immunodeficiency virus positive test. |
| 17 | Treatment with anti–T-cell antibody such as alemtuzumab (anti-CD52), excluding ATG (ATG-F or thymoglobulin), within 4 months before the first dose of study drug on day −1. |
| 18 | Treatment with any live vaccinations within 30 days before randomization. |
| 19 | Diagnosed or treated for another malignancy within 2 years before the first dose of study drug or previously diagnosed with another malignancy and have any evidence of residual disease. Patients with non-melanoma skin cancer or carcinoma in situ of any type are not excluded if they have undergone complete resection. |
| Excluded medications | |
| 1 | Any investigational agent (other than vedolizumab), including agents other than corticosteroids for treatment of GVHD. |
| 2 | Checkpoint inhibitors. |
| 3 | Any therapy for aGVHD prophylaxis other than that specified in the inclusion criteria. Should 1 of the GVHD prophylaxis agents specified in the inclusion criteria be discontinued due to toxicity and an alternative agent started, the patient may be permitted to remain on study treatment after consultation with the medical monitor. |
| 4 | All live vaccines from 30 days before screening to ≥6 months after the last dose of study drug. |
| 5 | Either approved or investigational monoclonal antibody or equivalent biologics for the treatment of other conditions (e.g., rheumatoid arthritis), other than localized injections (e.g., intraocular injections for wet macular degeneration). |

*aGVHD* acute graft-versus-host disease; *allo-HSCT* allogenic hematopoietic stem cell transplantation; *ATG* anti-thymocyte globulin; *CMV* cytomegalovirus; *CNI* calcineurin inhibitor; *CYS* cyclosporine; *ECOG* Eastern Cooperative Oncology Group; *GI* gastrointestinal; *GVHD* graft-versus-host disease; *HLA* human leukocyte antigen; *HSC* hematopoietic stem cells; *mAb* monoclonal antibody; *MAdCAM-1* mucosal vascular addressin cell adhesion molecule 1; *MMF* mycophenolate mofetil; *MTX* methotrexate; *PK* pharmacokinetic; *PML* progressive multifocal leukoencephalopathy; *RIC* reduced intensity conditioning; *TAC* tacrolimus.

1. Oken MM, Creech RH, Tormey DC, Horton J, Davis TE, McFadden ET, Carbone PP. Toxicity and response criteria of the Eastern Cooperative Oncology Group. Am J Clin Oncol. 1982;5(6):649–55. 2. Center for International Blood & Marrow Transplant Research. Forms Instruction Manual. Appendix L: Karnofsky/Lansky Performance Status. 2009. <https://cdn.manula.com/user/3235/3235_3360_3509_en_1418684645.pdf?v=20230828103227>.

**Supplementary Table S4** aGVHD clinical stage [1]

| Stage | Skin^a^ | Liver bilirubin, SI units  (standard units) | Intestinal tract^b^ (diarrhea/day) | |
| --- | --- | --- | --- | --- |
|  |  |  | Aged ≥18 years (or <18 years and ≥50 kg) | Aged <18 years and <50 kg |
| 1 | Maculopapular rash <25% of BSA | 34–50 µmol/L (2–3 mg/dL) | >500 mL diarrhea/day | 10–19.9 mL/kg/d |
| 2 | Maculopapular rash 25–50% of BSA | 51–102 µmol/L (3.1–6 mg/dL) | >1000 mL diarrhea/day | 20–30 mL/kg/d |
| 3 | Rash >50% of BSA | 103–255 µmol/L (6.1–1.5 mg/dL) | >1500 mL diarrhea/day | >30 mL/kg/d |
| 4 | Generalized erythroderma with bullous formation | >255 µmol/L (>15 mg/dL) | Severe abdominal pain, with or without ileus | Severe abdominal pain, with or without ileus |

*aGVHD* acute graft-versus-host disease; *BSA* body surface area.

^a^Use the “Rule of Nines” or burn chart to determine the extent of the rash.

^b^Staging of intestinal tract should be assessed based on patient’s age and weight at the time of assessment.

1. Przepiorka D, Weisdorf D, Martin P, Klingemann HG, Beatty P, Hows J, Thomas ED. 1994 Consensus Conference on Acute GVHD Grading. Bone Marrow Transplant. 1995;15(6):825–8.

**Supplementary Table S5** aGVHD grade (modified Glucksberg criteria) [1]

| Grade | Skin | Liver | Intestinal tract |
| --- | --- | --- | --- |
| I | Stage 1–2 | None | None |
| II | Stage 3 *or* → | Stage 1 *or* → | Stage 1 |
| III | – | Stage 2–3 *or* → | Stage 2–4 |
| IV | Stage 4 *or* → | Stage 4 | – |

*aGVHD* acute graft-versus-host disease.

1. Przepiorka D, Weisdorf D, Martin P, Klingemann HG, Beatty P, Hows J, Thomas ED. 1994 Consensus Conference on Acute GVHD Grading. Bone Marrow Transplant. 1995;15(6):825–8.

**Supplementary Table S6** Criteria for IBMTR Severity Index for aGVHD [1]

| Index | Skin | Liver | Intestinal tract |
| --- | --- | --- | --- |
|  | Stage (maximum) | Stage (maximum) | Stage (maximum) |
| A | Stage 1 | Stage 0 | Stage 0 |
| B | Stage 2 *or* | Stage 1–2 *or* | Stage 1–2 |
| C | Stage 3 *or* | Stage 3 *or* | Stage 3 |
| D | Stage 4 *or* | Stage 4 *or* | Stage 4 |

*aGVHD* acute graft-versus-host disease; *IBMTR* International Bone Marrow Transplant Registry.

1. Rowlings PA, Przepiorka D, Klein JP, Gale RP, Passweg JR, Henslee-Downey PJ, Cahn JY, Calderwood S, Gratwohl A, Socié G, Abecasis MM, Sobocinski KA, Zhang MJ, Horowitz MM. IBMTR Severity Index for grading acute graft-versus-host disease: retrospective comparison with Glucksberg grade. Br J Haematol. 1997;97(4):855–64. <https://doi.org/10.1046/j.1365-2141.1997.1112925.x>.

**Supplementary Table S7** Criteria for MAGIC Severity Index for aGVHD [1]

| Stage* | Skin (active erythema only) | Liver (bilirubin) | Upper GI | Intestinal tract (stool output/day) | |
| --- | --- | --- | --- | --- | --- |
|  |  |  |  | Aged ≥18 years (or <18 years and ≥50 kg) | Aged <18 years and <50 kg |
| 0 | No active (erythematous) GVHD rash | <2 mg/dL | No or intermittent nausea, vomiting, or anorexia | <500 mL/day or <3 episodes/day | <10 mL/kg/day or <4 episodes/day |
| 1 | Maculopapular rash <25% of body surface | 2–3 mg/dL | Persistent nausea, vomiting, or anorexia | 500–999 mL/day or 3–4 episodes/day | 10–19.9 mL/kg/day or 4–6 episodes/day |
| 2 | Maculopapular rash 25–50% of body surface | 3.1–6 mg/dL | – | 1000–1500 mL/day or 5–7 episodes/day | 20–30 mL/kg/day or 7–10 episodes/day |
| 3 | Maculopapular rash >50% of body surface | 6.1–15 mg/dL | – | >1500 mL/day or >7 episodes/day | >30 mL/kg/day or >10 episodes/day |
| 4 | Generalized erythroderma (>50% BSA) + bullous formation and desquamation >5% body surface | >15 mg/dL | – | Severe abdominal pain, with or without ileus, or grossly bloody stool (regardless of stool volume) | Severe abdominal pain, with or without ileus, or grossly bloody stool (regardless of stool volume) |

*aGVHD* acute graft-versus-host disease; *BSA* body surface area; *GI* gastrointestinal; *GVHD* graft-versus-host disease; *MAGIC* Mount Sinai Acute GVHD International Consortium.

^a^Overall clinical grade (based on most severe target organ involvement): Grade 0, no Stage 1–4 of any organ; Grade I, Stage 1–2 skin without liver, upper-GI, or lower-GI involvement; Grade II, Stage 3 rash and/or Stage 1 liver and/or Stage 1 upper GI and/or Stage 1 lower GI; Grade III, Stage 2–3 liver and/or Stage 2–3 lower GI, with Stage 0–3 skin and/or Stage 0–1 upper GI; Grade IV, Stage 4 skin, liver or lower-GI involvement with Stage 0–1 upper GI.

^b^Staging of intestinal tract should be assessed based on patient’s age and weight at the time of assessment.

1. Harris AC, Young R, Devine S, Hogan WJ, Ayuk F, Bunworasate U, Chanswangphuwana C, Efebera YA, Holler E, Litzow M, Ordemann R, Qayed M, Renteria AS, Reshef R, Wölfl M, Chen YB, Goldstein S, Jagasia M, Locatelli F, Mielke S, Porter D, Schechter T, Shekhovtsova Z, Ferrara JL, Levine JE. International, multicenter standardization of acute graft-versus-host disease clinical data collection: a report from the Mount Sinai Acute GVHD International Consortium. Biol Blood Marrow Transplant. 2016;22(1):4–10. https://doi.org/10.1016/j.bbmt.2015.09.001.

**Supplementary Table S8** Patient disposition.

| Randomized patients | Japanese (n = 37) | | | Non-Japanese (n = 306) | | |
| --- | --- | --- | --- | --- | --- | --- |
|  | PBO (n = 18) | VDZ (n = 19) | Total (N = 37) | PBO (n = 152) | VDZ (n = 154) | Total (N = 306) |
| Received study treatment, n | 17 | 19 | 36 | 148 | 150 | 298 |
| Randomized, not treated, n (%) | 0 | 1 (5.0) | 1 (2.7) | 4 (2.6) | 4 (2.6) | 8 (2.6) |
| Discontinued study treatment, n (%) | 5 (29.4) | 8 (40.0) | 13 (35.1) | 76 (50.0) | 58 (37.7) | 134 (43.8) |
| Adverse event | 1 (20.0) | 5 (62.5) | 6 (46.2) | 20 (26.3) | 18 (31.0) | 38 (28.4) |
| Protocol deviation | 0 | 1 (12.5) | 1 (7.7) | 5 (6.6) | 1 (1.7) | 6 (4.5) |
| Withdrawal by patient | 0 | 1 (12.5) | 1 (7.7) | 9 (11.8) | 7 (12.1) | 16 (11.9) |
| Unsatisfactory therapeutic response | 1 (20.0) | 1 (12.5) | 2 (15.4) | 21 (27.6) | 10 (17.2) | 31 (23.1) |
| Death | 2 (40.0) | 0 | 2 (15.4) | 17 (22.4) | 16 (27.6) | 33 (24.6) |
| Other | 1 (20.0) | 0 | 1 (7.7) | 3 (3.9) | 4 (6.9) | 7 (5.2) |
| Other (COVID-19 related) | 0 | 0 | 0 | 1 (1.3) | 2 (3.4) | 3 (2.2) |
| Completed day +180 visit, n (%) | 14 (82.4) | 19 (95.0) | 33 (89.2) | 119 (78.3) | 130 (84.4) | 249 (81.4) |
| Completed the study, n (%) | 10 (58.8) | 15 (75.0) | 25 (67.6) | 88 (57.9) | 102 (66.2) | 190 (62.1) |
| Discontinued the study, n (%) | 7 (41.2) | 5 (25.0) | 12 (32.4) | 64 (42.1) | 52 (33.8) | 116 (37.9) |
| Adverse event | 0 | 1 (20.0) | 1 (8.3) | 5 (7.8) | 5 (9.6) | 10 (8.6) |
| Protocol deviation | 0 | 0 | 0 | 3 (4.7) | 0 | 3 (2.6) |
| Withdrawal by patient | 3 (42.9) | 2 (40.0) | 5 (41.7) | 15 (23.4) | 14 (26.9) | 29 (25.0) |
| Unsatisfactory therapeutic response | 0 | 1 (20.0) | 1 (8.3) | 5 (7.8) | 2 (3.8) | 7 (6.0) |
| Study termination | 0 | 0 | 0 | 1 (1.6) | 0 | 1 (0.9) |
| Death | 4 (57.1) | 0 | 4 (33.3) | 29 (45.3) | 26 (50.0) | 55 (47.4) |
| COVID-19–related death | 0 | 0 | 0 | 1 (1.6) | 0 | 1 (0.9) |
| Other | 0 | 1 (20.0) | 1 (8.3) | 5 (7.8) | 4 (7.7) | 9 (7.8) |
| Other (COVID-19 related) | 0 | 0 | 0 | 0 | 1 (1.9) | 1 (0.9) |

*PBO* placebo; *VDZ* vedolizumab.

**Supplementary Table S9** Frequency of aGVHD by organ involvement and maximum clinical stage

| Day +180 after allo-HSCT | Japanese | | Non-Japanese | |
| --- | --- | --- | --- | --- |
|  | PBO (n = 17) | VDZ (n = 18) | PBO (n = 148) | VDZ (n = 150) |
| aGVHD of the lower-GI tract, n (%) | | | | |
| Stage 1 | 0 | 1 (5.6) | 11 (7.6) | 5 (3.4) |
| Stage 2 | 1 (5.9) | 0 | 1 (0.7) | 2 (1.3) |
| Stage 3 | 0 | 0 | 9 (6.3) | 1 (0.7) |
| Stage 4 | 0 | 0 | 3 (2.1) | 1 (0.7) |
| aGVHD of the skin, n (%) | | | | |
| Stage 1 | 6 (35.3) | 6 (33.3) | 17 (11.6) | 28 (18.8) |
| Stage 2 | 1 (5.9) | 2 (11.1) | 24 (16.3) | 20 (13.4) |
| Stage 3 | 3 (17.6) | 2 (11.1) | 21 (14.3) | 17 (11.4) |
| Stage 4 | 0 | 0 | 4 (2.7) | 2 (1.3) |
| aGVHD of the liver, n (%) | | | | |
| Stage 1 | 0 | 0 | 2 (1.4) | 3 (2.0) |
| Stage 2 | 0 | 1 (5.6) | 2 (1.4) | 0 |
| Stage 3 | 0 | 0 | 2 (1.4) | 0 |
| Stage 4 | 0 | 0 | 0 | 0 |

.

| Day +365 after allo-HSCT | Japanese | | Non-Japanese | |
| --- | --- | --- | --- | --- |
|  | PBO (n = 17) | VDZ (n = 18) | PBO (n = 148) | VDZ (n = 150) |
| aGVHD of the lower-GI tract, n (%) | | | | |
| Stage 1 | 0 | 1 (5.6) | 11 (7.6) | 5 (3.4) |
| Stage 2 | 1 (5.9) | 0 | 2 (1.4) | 2 (1.3) |
| Stage 3 | 0 | 1 (5.6) | 10 (6.9) | 3 (2.0) |
| Stage 4 | 0 | 0 | 3 (2.1) | 2 (1.3) |
| aGVHD of the skin, n (%) | | | | |
| Stage 1 | 6 (35.3) | 6 (33.3) | 19 (12.9) | 31 (20.8) |
| Stage 2 | 1 (5.9) | 3 (16.7) | 24 (16.3) | 22 (14.8) |
| Stage 3 | 3 (17.6) | 2 (11.1) | 21 (14.3) | 19 (12.8) |
| Stage 4 | 0 | 0 | 4 (2.7) | 2 (1.3) |
| aGVHD of the liver, n (%) | | | | |
| Stage 1 | 0 | 0 | 2 (1.4) | 3 (2.0) |
| Stage 2 | 0 | 1 (5.6) | 2 (1.4) | 0 |
| Stage 3 | 0 | 0 | 2 (1.4) | 1 (0.7) |
| Stage 4 | 0 | 0 | 0 | 0 |

*aGVHD* acute graft-versus-host disease; *allo-HSCT* allogeneic hematopoietic stem cell transplant; *PBO* placebo; *VDZ* vedolizumab

**Supplementary** **Table S10** Key exploratory endpoint results in Japanese and non-Japanese patients

| Key exploratory endpoints (by day +365) | Japanese | | | | Non-Japanese | | | |
| --- | --- | --- | --- | --- | --- | --- | --- | --- |
|  | PBO (n = 17) | VDZ (n = 18) | VDZ vs PBO | | PBO (n = 148) | VDZ (n = 150) | VDZ vs PBO | |
|  | Events, n (%) | | P value^a^ | HR (95% CI)^b^ | Events, n (%) | | P value^a^ | HR (95% CI)^b^ |
| **1.** Lower Gl aGVHD-free survival | | | | | | | | |
| Events of lower-GI aGVHD, or death | 4 (23.5) | 2 (11.1) | 0.2844 | 0.53 (0.09–3.22) | 52 (35.1) | 34 (22.7) | 0.0075* | 0.55 (0.36–0.86) |
| **2.** IBMTR^c^ Grade C–D aGVHD-free (any organ involvement) survival | | | | | | | | |
| Events of Grade C–D aGVHD or death | 5 (29.4) | 3 (16.7) | 0.2397 | 0.55 (0.12–2.51) | 54 (36.5) | 44 (29.3) | 0.1228 | 0.71 (0.48–1.07) |
| **3.** Non-relapse mortality | | | | | | | | |
| Events of death without relapse | 3 (17.6) | 1 (5.6) | 0.2311 | 0.39 (0.03–4.44) | 22 (14.9) | 14 (9.3) | 0.1267 | 0.54 (0.27–1.09) |
| **4.** Overall survival | 4 (23.5) | 1 (5.6) | 0.1146 | 0.26 (0.03–2.50) | 32 (21.6) | 27 (18.0) | 0.3466 | 0.76 (0.45–1.28) |
| **5.** IBMTR^c^ Grade B–D aGVHD-free (any organ involvement) survival | | | | | | | | |
| Events of Grade B–D aGVHD or death | 6 (35.3) | 6 (33.3) | 0.6709 | 0.98 (0.29–3.27) | 76 (51.4) | 63 (42.0) | 0.0622 | 0.72 (0.51–1.01) |
| **6.** GVHD (any organ)-free and relapse (of the underlying malignancy)-free survival | | | | | | | | |
| Events of aGVHD Grade 3–4 by modified Glucksberg or chronic GVHD requiring systemic immunosuppression or death | 5 (29.4) | 3 (16.7) | 0.2967 | 0.61 (0.14–2.76) | 59 (39.9) | 53 (35.3) | 0.2616 | 0.83 (0.57–1.21) |
| **7.** Progression-free survival | | | | | | | | |
| Events of relapse (of the underlying malignancy) or death | 4 (23.5) | 3 (16.7) | 0.5564 | 0.83 (0.17–4.17) | 43 (29.1) | 45 (30.0) | 0.9971 | 1.01 (0.67–1.55) |

*aGVHD* acute graft-versus-host disease; *CI* confidence interval; *GI* gastrointestinal; *HR* hazard ratio; *IBMTR* International Bone Marrow Transplant Register; *PBO* placebo; *VDZ* vedolizumab.
*Statistically significant for full analysis set: all patients who received ≥1 dose of study treatment and received allogenic hematopoietic stem cell transplantation.
^a^P value was obtained from log-rank tests.

^b^HRs and 95% CIs were obtained from Cox proportional hazards models with treatment group stratified by randomization strata: human leukocyte antigen match (7/8, 8/8), conditioning regimen (myeloablative conditioning, reduced intensity conditioning), and anti-thymocyte globulin (with, without).

^c^IBMTR Severity Index for aGVHD Grade C–D is equivalent to maximum Stage 3–4 skin or liver or GI tract, Grade B–D is maximum Stage 2–4 skin or Stage 1–2 to 4 liver or Stage 1–2 to 4 GI tract (adapted from Rowlings et al. *Br J Haematol* 1997;97:855-64).

**Supplementary** **Table S11** Colitis adverse events

| Patients, n (%) | Japanese | | Non-Japanese | |
| --- | --- | --- | --- | --- |
|  | PBO (n = 17) | VDZ (n = 19) | PBO (n = 148) | VDZ (n = 150) |
| Enterocolitis | 1 (5.9) | 0 | 1 (0.7) | 2 (1.3) |
| Enterocolitis infections | 0 | 0 | 1 (0.7) | 3 (2.0) |
| *Clostridioides difficile* colitis | 1 (5.9) | 0 | 2 (1.4) | 4 (2.7) |
| *Clostridioides* colitis | 0 | 0 | 1 (0.7) | 0 |
| Colitis | 0 | 0 | 5 (3.4) | 0 |
| Neutropenic colitis | 0 | 0 | 1 (0.7) | 2 (1.3) |
| Cytomegalovirus colitis | 0 | 0 | 1 (0.7) | 1 (0.7) |

*PBO* placebo; *VDZ* vedolizumab.

**Supplementary Figure S1.**

Kaplan-Meier curves for key secondary endpoints in Japanese patients. Full analysis set: all patients who received ≥1 dose of study treatment and received allo-HSCT from the Japanese cohort. P values were obtained from log-rank tests. Key secondary efficacy endpoints were tested following a fixed-sequence hierarchical testing procedure. When 1 efficacy endpoint was found not significant (i.e., P > 0.05), testing of all subsequent endpoints would not be performed. HRs and 95% CIs were obtained from Cox proportional hazards models with treatment group stratified by randomization strata: HLA match (7/8, 8/8), conditioning regimen (MAC, RIC), and ATG (with, without). ^a^IBMTR Severity Index for aGVHD Grade C–D is equivalent to maximum Stage 3–4 skin or liver or GI tract, Grade B–D is maximum Stage 2–4 skin or Stage 1–2 to 4 liver or Stage 1–2 to 4 GI tract (adapted from Rowlings et al. *Br J Haematol* 1997;97:855-64). *IBMTR* International Bone Marrow Transplant Register; *NE* not evaluable; *PBO* placebo; *VDZ* vedolizumab.


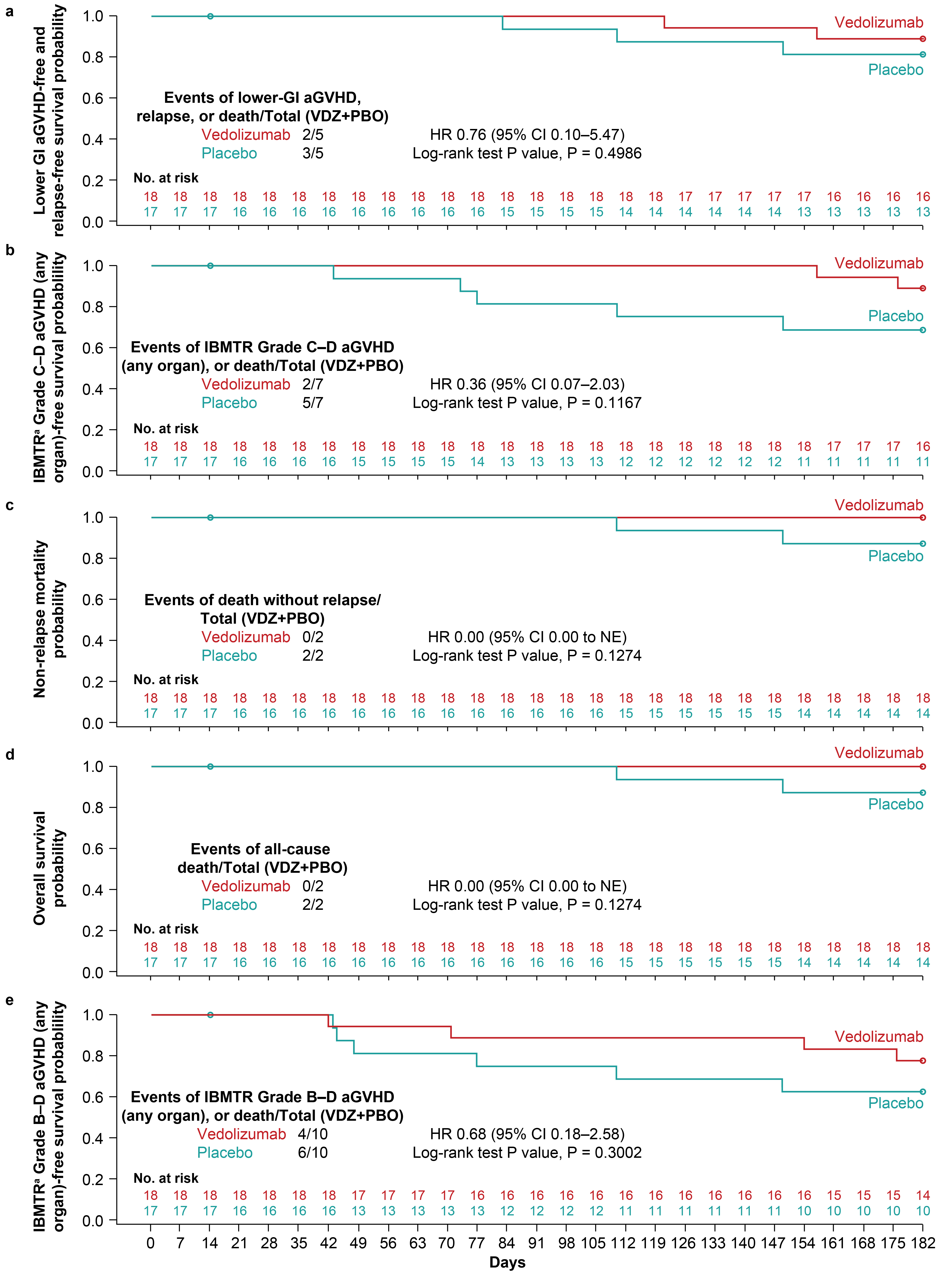


**Supplementary Figure S2.**

Kaplan-Meier curves for key secondary endpoints in non-Japanese patients. Full analysis set: all patients who received ≥1 dose of study treatment and received allo-HSCT from the non-Japanese cohort. P values were obtained from log-rank tests. Key secondary efficacy endpoints were tested following a fixed-sequence hierarchical testing procedure. When 1 efficacy endpoint was found not significant (i.e., P > 0.05), testing of all subsequent endpoints would not be performed. HRs and 95% CIs were obtained from Cox proportional hazards models with treatment group stratified by randomization strata: HLA match (7/8, 8/8), conditioning regimen (MIC, RIC), and ATG (with, without). ^a^IBMTR Severity Index for aGVHD Grade C–D is equivalent to maximum Stage 3–4 skin or liver or GI tract, Grade B–D is maximum Stage 2–4 skin or Stage 1–2 to 4 liver or Stage 1–2 to 4 GI tract (adapted from Rowlings et al. *Br J Haematol* 1997;97:855-64). *IBMTR* International Bone Marrow Transplant Register; *PBO* placebo; *VDZ* vedolizumab.


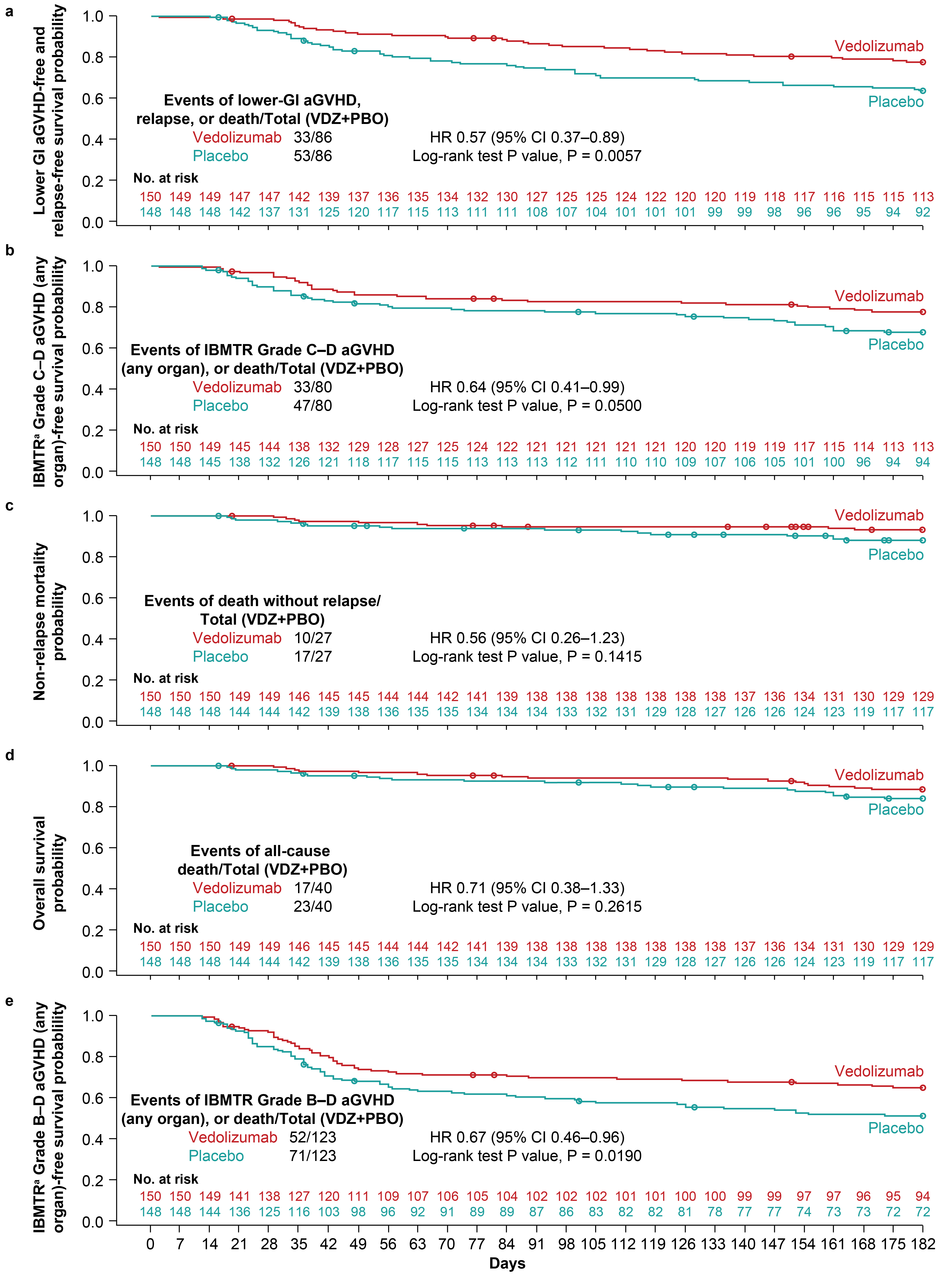

Supplement: Supplementary file 1 — Supplementary file1 (DOCX 7003 KB) [file 12185_2025_3955_MOESM1_ESM.docx]
